# Brain metastasis from hepatocellular carcinoma: the role of surgery as a prognostic factor

**DOI:** 10.1186/1471-2407-13-567

**Published:** 2013-12-01

**Authors:** Moon-Soo Han, Kyung-Sub Moon, Kyung-Hwa Lee, Sung-Bum Cho, Sa-Hoe Lim, Woo-Youl Jang, Tae-Young Jung, In-Young Kim, Shin Jung

**Affiliations:** 1 Department of Neurosurgery, Chonnam National University Research Institute of Medical Sciences, Chonnam National University Hwasun Hospital & Medical School, Gwangju, South Korea; 2 Department of Pathology, Chonnam National University Research Institute of Medical Sciences, Chonnam National University Hwasun Hospital & Medical School, Gwangju, South Korea; 3 Department of Internal Medicine, Chonnam National University Research Institute of Medical Sciences, Chonnam National University Hwasun Hospital & Medical School, Gwangju, South Korea

**Keywords:** Brain metastasis, Hepatocellular carcinoma, Prognosis, Surgical resection, Survival

## Abstract

**Background:**

The incidence of brain metastasis from hepatocellular carcinoma (HCC) is expected to increase as a result of prolonged survival due to the recent advances in HCC treatment. However, there is no definite treatment strategy for brain metastasis from HCC mainly due to its rarity and dismal prognosis. To provide helpful recommendations in treatment of brain metastasis from HCC, the authors aimed to identify prognostic factors that influence survival rates with a review of the recently published data.

**Methods:**

Thirty-three cases of brain metastasis, whose incidence was 0.65%, were selected from a total of 5015 HCC patients and reviewed retrospectively in terms of clinical and radiological features.

**Results:**

Median overall survival time after diagnosis of brain metastasis was 10.4 weeks (95% confidence interval [CI], 5.1-15.7 weeks) with 1-, 6- and 12-month survival rates, of 79%, 24% and 6%, respectively. Median survival of the patients treated with surgical resection or surgical resection followed by whole-brain radiation therapy (WBRT) (25.3 weeks; range, 15.8-34.8 weeks) was longer than that of the patients treated with gamma knife surgery (GKS), WBRT, or GKS followed by WBRT (10.4 weeks; range, 7.5-13.3 weeks) as well as that of patients treated with only steroids (1 week; range, 0.0-3.3 weeks) (p < 0.001). Child-Pugh’s classification A group had a longer median survival time than Child-Pugh’s classification B or C group (14.4 weeks vs 8.4 weeks, p = 0.038). RPA class I & II group had also a longer median survival time than RPA class III group did (13.4 weeks *vs* 2.4 weeks, p = 0.001). Surgical resection (hazard ratio [HR] 0.23, 95% CI 0.08-0.66, p = 0.006) and good liver function at the time of brain metastasis (HR 0.25, 95% CI 0.09-0.69, p = 0.007) were found to be the powerful prognostic factors for favorable survival in the multivariate analysis. In addition, presence of intratumoral hemorrhage was a statistically significant prognostic factor for survival.

**Conclusion:**

Although HCC patients with brain metastasis showed a very dismal prognosis, surgical intervention was shown to lead to relative prolongation of the survival time, especially in those with preserved hepatic function.

## Background

Hepatocellular carcinoma (HCC) is one of the most common malignant tumors worldwide [1]. Its incidence is particularly high in Southeast Asia and sub-Saharan Africa where hepatitis B and C infections are the most prevalent [1]; however, a significant increase in the incidence of HCC has been recently observed in Australia and other Western countries [1]. Brain metastasis from HCC is so rare that the incidence was reported to be only about 0.6% [2]. The recent therapeutic advances including surgical techniques, transarterial chemoembolization (TACE), local ablation, and chemotherapeutic agents, have all contributed to improved survival rates [3]. The incidence of brain metastasis, therefore, is expected to increase as a result of prolonged survival of HCC patients [4]. The prognosis for patients having brain metastasis from HCC is very poor [5-7]. There have been no definite recommendations for the management of the brain metastasis from HCC, because of its rarity and poor prognosis.

In the present study, the authors aimed to elucidate the incidence of brain metastasis from HCC and to identify prognostic factors that influence survival rates. In addition, we reviewed the literature to identify prognostic factors for survival in patients with brain metastasis from HCC.

## Methods

The study is in compliance with the Declaration of Helsinki (Sixth Revision, 2008). This study fulfills all the requirements for patient anonymity was approved by the institutional review board of Chonnam National University Medical School Research Institution (2013–67). Of a total 5015 HCC patients who were diagnosed and treated at our hospital between 2001 and 2012, 33 patients were retrospectively confirmed as having brain metastasis from HCC, using cranial computed tomography (CT) and/or magnetic resonance imaging (MRI) and by reviewing the hospital charts.

To define the clinical characteristics of the patients with brain metastasis from HCC, clinical data at the time of diagnosis of brain metastasis, including age, sex, presenting symptoms, time interval from diagnosis of primary tumor to brain metastasis, Eastern cooperative oncology group (ECOG) performance status [8], Child-Pugh classification [9], recursive partitioning analysis (RPA) class [10], level of alpha fetoprotein (AFP), treatment modality, and survival time were collected. Additionally, we also evaluated the radiological findings such as the presence of extracranial metastasis and main portal vein thrombosis, number and type of HCC nodule, size of the largest HCC nodule, number of brain lesions, concomitant intratumoral hemorrhagic changes and location of the brain lesion.

ECOG performance status described six grades to determine the appropriate treatment and prognosis [8]: Grade 0: Fully active, able to carry on all pre-disease performance; Grade 1: Restricted in physically strenuous activity but ambulatory and able to carry out work of a light or sedentary nature; Grade 2: Ambulatory and capable of all self-care but unable to carry out any work activities, Up and about more than 50% of waking hours; Grade 3: Capable of only limited self-care, confined to bed or chair more than 50% of waking hours; Grade 4: Completely disabled. Cannot carry on any self-care. Totally confined to bed or chair; Grade 5: Dead.

Child-Pugh classification [9] includes the factors of hepatic encephalopathy, ascites, total bilirubin level, albumin level, and prolonged prothrombin time. Child-Pugh A category is defined as a score of 5–6, Child-Pugh B category is defined as a score of 7–9, and Child-Pugh C category is defined as a score of 10–15. Overall survival was calculated from the date of diagnosis of brain metastasis until death, or until the date of the last follow-up visit for patients who were still alive.

RPA classification describes three classes to predict survival of patients with brain metastases [10]: Class I: patients with a Karnofsky performance status (KPS) > 70, age < 65 years with controlled primary disease and no evidence of extracranial metastases; Class III: patients with a KPS < 70, and Class II: all remaining patients who do not fit into Classes 1 or III. Stratified Mantel-Cox log-rank test for each factor was applied to compare the Kaplan-Meier curves for survival. The factors that might predict overall survival in HCC patients with brain metastasis were analyzed using a multivariate logistic regression model. All statistical analyses were performed using SPSS version 20.0 software program for Windows (SPSS, Chicago, IL, USA). The level of significance was set at P < 0.05.

## Results

### Clinical presentation

The incidence of brain metastasis from HCC was 0.65% in our patient group (33/5015). Clinical characteristics of the enrolled patients are summarized in Table 1. The median age at diagnosis of brain metastasis was 62 years (range, 23–80 years) and there was a male predominance (91%). The most common presenting symptoms were headache, followed by motor weakness and mental status changes. The majority of the lesions were symptomatic (97%), possibly due to the high incidence of intratumoral hemorrhage (52%). The median time interval between diagnosis of HCC and diagnosis of brain metastasis was 18.3 months (range, 0.5-75 months). In RPA classification at the time of diagnosis of brain metastasis, the majority of the patients were grouped into class II (27 patients, 82%) or III (4 patients, 12%). According to Child-Pugh classification for the severity of liver function, 20 patients (61%) were classified into grade A, 13 patients (39%) into grade B or C. Elevated AFP levels were found in only 10 patients (30%). Viral hepatitis infection was detected in most of the patients (85%) on serological study with a high prevalence of hepatitis B virus infection (76%). In the aspect of HCC characteristics, only 8 patients (24%) showed smaller number of nodule lesser than 4 (<4). The maximum diameter of the HCC nodule was greater than 5 cm in 15 patients (45%). Ill-defined types of HCC were found in 13 patients (39%) and thrombosis of the main portal vein was present in 15 patients (45%). Chemotherapy using a target agent (Sorafenib®) was applied in 10 patients (30%).

**Table 1:** Clinical characteristics of 33 patients with brain metastases from HCC

**Characteristic**	**No. of patients (%)**
**HCC characteristics**	
Age in years at diagnosis of brain metastasis; median (range)	62 (23-80yrs)
Sex	
Male	30 (91%)
Female	3 (9%)
Time in months from diagnosis of HCC to brain metastases; median (range)	18.3 (0.5-75mos)
ECOG performance status	
≤2	21 (64%)
≥3	12 (36%)
RPA class I	2 (6%)
II	27 (82%)
III	4 (12%)
Child-Pugh classification	
A	20 (61%)
B	11 (33%)
C	2 (6%)
AFP (ng/ml)	
≤400	18 (55%)
>400	10 (30%)
unknown	5 (15%)
Etiology	
Hepatitis B	25 (76%)
Hepatitis C	3 (9%)
Alcoholic	4 (12%)
Idiopathic	1 (3%)
Number of tumor nodules	
1	3 (9%)
2-3	5 (15%)
≥4	25 (76%)
Largest tumor size (cm)	
<2	4 (12%)
2-5	14 (43%)
6-10	12 (36%)
>10	3 (9%)
Tumor type	
Well-defined	20 (61%)
Ill-defined	13 (39%)
Main portal vein thrombosis	
Absent	18 (55%)
Present	15 (45%)
Previous treatment	
Hepatic resection	5 (15%)
TACE	27 (82%)
RFA	4 (12%)
Chemotherapy (Sorafenib)	10 (30%)
Radiotherapy	10 (30%)
Site of extracranial metastases	
Lung	24 (73%)
Bone	6 (18%)
Lymph node	8 (24%)
Adrenal gland	2 (6%)
Skin	1 (3%)
None	6 (18%)
**Brain metastasis characteristics**	
Symptoms/signs	
Headache	18 (55%)
Motor disturbance	12 (36%)
Mental status changes	8 (24%)
Visual disturbance	5 (15%)
Dizziness	4 (12%)
Non-neurologic symptom	1 (3%)
Number of lesions	
Single	17 (52%)
Multiple	16 (48%)
Intratumoral hemorrhage	
Yes	17 (52%)
No	16 (48%)
Location of brain metastases	
Frontal	5 (15%)
Parietal	6 (18%)
Occipital	2 (6%)
Temporal	1 (3%)
Cerebellar	1 (3%)
Cavernous sinus	1 (3%)
Basal ganglia	1 (3%)
Multiple locations	16 (48%)
Treatment modalities	
Resection alone	4 (12%)
Resection + WBRT	6 (18%)
GKS alone	11 (33%)
GKS + WBRT	2 (6%)
WBRT alone	4 (12%)
Palliative (Steroid alone)	6 (18%)

Twenty-four patients (73%) had lung metastasis at the time of detection of brain metastasis. Seventeen patients (52%) had a single brain lesion.

### Treatment

Accordingly, treatment methods were decided based on many factors, including general condition of the patient, the number and location of brain lesions, physician’s and patients’ personal preference. Treatment modalities for brain metastasis are summarized in Table 1. Ten patients underwent surgical resection and six of them subsequently received WBRT. Thirteen patients were treated with GKS, and four patients were treated with WBRT alone. Two patients who were previously treated with GKS showed tumor recurrence, and hence additional WBRT was performed in these patients. Six patients received conservative medical (palliative) treatment, mainly with steroids to reduce the increased intracranial pressure. All operation cases underwent gross total resection. Mean maximal and marginal doses of GKS were 36.4 Gy (range, 24–50 Gy) and 18 Gy (range, 14–25 Gy) prescribed to the 50% iso-dose line. The usual fractionation schedule for WBRT was 30 Gy in 10 fractions (Mean dose, 28.8 Gy ; range, 25–30 Gy).

### Overall survival and prognostic factors

**Figure 1:**
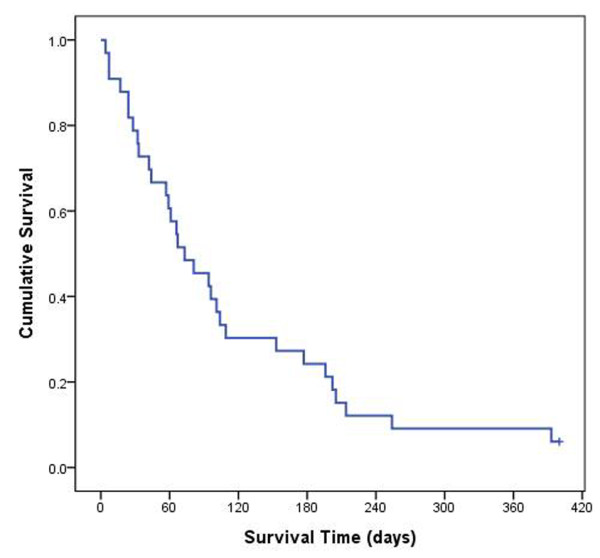
**Overall survival in 33 patients after the diagnosis of brain metastasis from HCC.** Note that the median survival was 10.4 weeks and 1-, 6- and 12-month survival rates were 79%, 24% and 6%, respectively.

Patients with brain metastasis from HCC had poor outcomes. The median overall survival time after diagnosis of brain metastasis was 10.4 weeks (Figure 1. 95% confidence interval [CI]: 5.1-15.7 weeks). The 1-, 6- and 12-month survival rates were 79%, 24% and 6%, respectively. At the end of the follow up, 31 patients had died and two patients were alive (one in radiation treatment group and one resection group). The cause of death was identified in 29 patients (not defined in two cases; one in radiation treatment group and one in resection group). Seventeen patients died as a result of progressive diseases or systemic complications (hepatic failure, acute respiratory failure, and so on), and 12 patients died as a consequence of metastatic brain diseases. With palliative treatment using steroid, 5 patient died due to brain lesions (5/6, 83%). In the radiation treatment group, causes of death were neurologic-origin in 6 patients (6/15, 40%). Only one patients (1/8, 13%) died from neurologic cause (rebleeding on another small lesion) in the surgical resection group.

**Figure 2:**
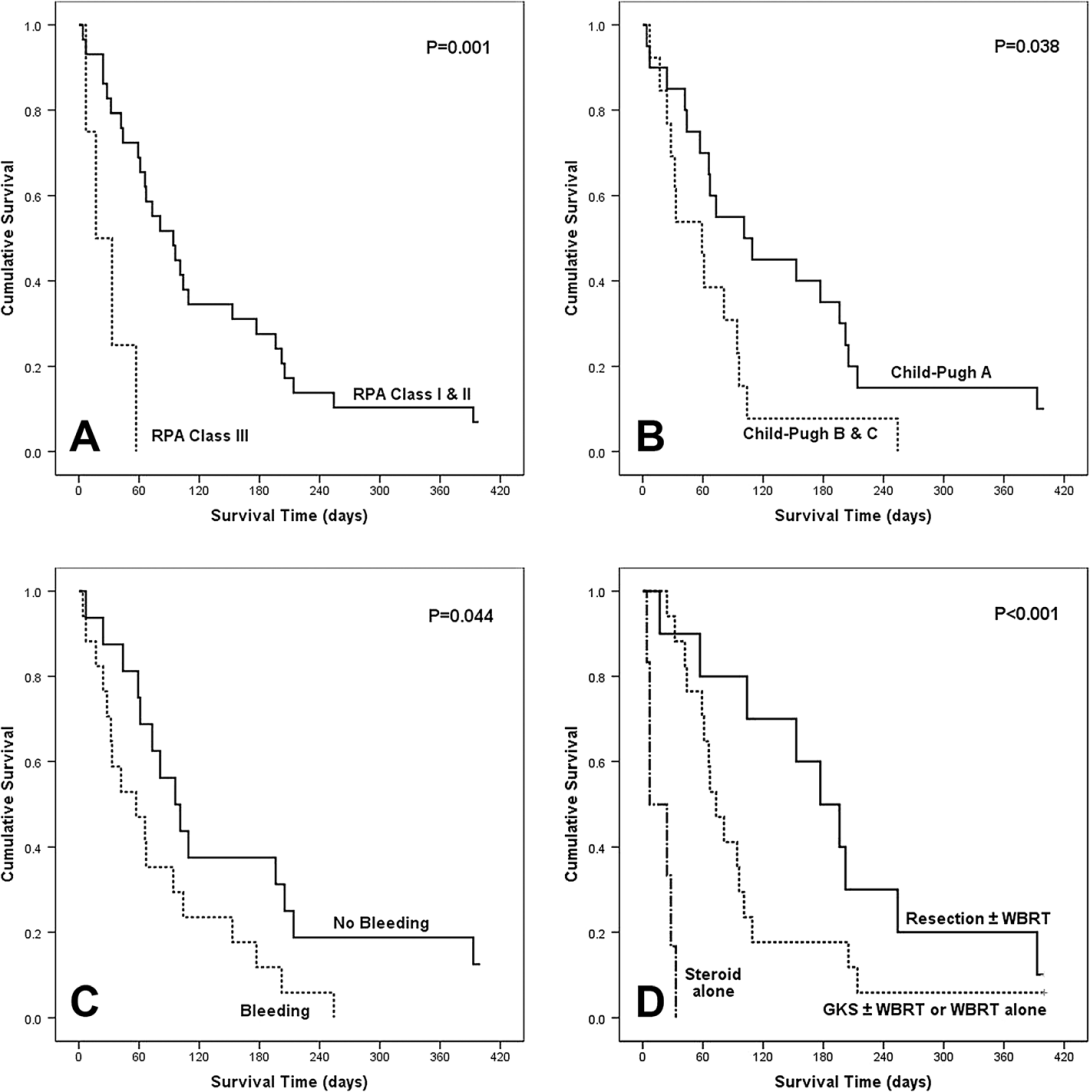
**Kaplan-Meier analyses of overall survival for 33 patients according to different predictors.** (overall comparison was estimated using a log-rank test). **A**: RPA class, **B**: Child-Pugh classification, **C**: Presence of intratumoral hemorrhage, **D**: Treatment modality for brain metastasis.

The results of analyses of the variables that could be correlated with survival time are shown in Table 2. On univariate analysis, RPA classification, Child-Pugh’s classification, intratumoral hemorrhage, and treatment modality showed statistical significance (Figure 2). The patients with RPA class I or II showed a longer survival time than the patients with RPA class III did, with a statistical significance in univariate analysis (13.4 weeks *vs* 2.4 weeks, p = 0.001). In multivariate analysis, however, this difference was not statistically significant (p = 0.063).

**Table 2:** Univariate and multivariate analyses for survival predictors in patients with brain metastasis from HCC

**Variables**		**No**	**Median(wks)**	**Univariate**	**Multivariate**		
				***p*-value**	**HR**	**95% CI**	***p*-value**
AGE				0.096	ND		
	<60 years	17	14.8				0.062
	≥60 years	16	8.7				
Sex				0.178	ND		
	M	30	10.4				0.144
	F	3	8.4				
Symptoms^#^				0.412	ND		
	Minor	17	11.6				0.552
	Major	16	8.1				
Interval of diagnosis from primary tumor to brain metastasis							
	≤12 months	11	15.6	0.239	ND		0.089
	>12 months	22	8.7				
ECOG PS				0.655	ND		
	≤2	21	13.4				0.707
	>2	12	4.7				
RPA class				0.001	ND		
	I & II	29	13.4				0.575
	III	4	2.4				
Child-Pugh’s classification							
	A	20	14.4	0.038	0.25	0.09-0.69	0.007
	B/C	13	8.4		1		
Number of HCC nodules							
	1-3	18	9.6	0.389	ND		0.088
	≥4	10	8.7				
Largest size of HCC							
	≤5 cm	18	14.4	0.094	ND		0.119
	>5 cm	15	6.0				
HCC type							
	Well-defined	20	12.9	0.210	ND		0.072
	Ill-defined	13	8.7				
Main portal vein thrombosis							
	No	18	10.4	0.202	ND		0.065
	Yes	15	8.7				
Chemotherapy for HCC (Sorafenib)							
	No	23	9.6	0.735	ND		0.583
	Yes	10	10.4				
AFP							
	≤400	18	9.6	0.389	ND		0.174
	>400	10	8.7				
Lung metastasis							
	No	9	21.9	0.197	ND		0.956
	Yes	24	9.4				
Number of brain metastasis							
	Single	17	13.7	0.341	ND		0.800
	Multiple	16	8.1				
Hemorrhage of brain metastasis							
	No	16	13.7	0.044	0.19	0.07-0.55	0.002
	Yes	17	8.1		1		
Treatment for brain metastasis							
	Resection ± WBRT	10	25.3	<0.001	0.23	0.08-0.66	0.006
	GKS± WBRT /WBRT alone	17	10.4		1^*^		
	Steroid alone	6	1.0				

# Minor; headache, dizziness, cranial nerve deficit, Major; mental status changes, motor weakness.

* Reference variable: non-resection treatment groups.

HCC; hepatocellular carcinoma, wks; weeks, AFP; alpha fetoprotein, WBRT; whole brain radiation therapy, GKS; gamma knife radiosurgery, ND; not determined.

Child-Pugh’s classification A group had a longer median survival time than Child-Pugh’s classification B or C group (14.4 weeks *vs* 8.4 weeks, p = 0.038). The statistical significance remained meaningful in multivariate analysis (HR 0.25, 95% CI 0.09-0.69, p = 0.007). Patients without intratumoral hemorrhage had a longer median survival time than patients with intratumoral hemorrhage (13.7 weeks *vs* 8.1 weeks, p = 0.044). Furthermore, this difference reached statistical significance in multivariate analysis (HR 0.19, 95% CI 0.07-0.55, p = 0.002). With respect to the treatment modality for brain metastasis, surgical treatment was shown to lead to a longer survival time in univariate analysis (p < 0.001); Median survival was 25.3 weeks (range, 15.8-34.8 weeks) for patients treated with surgical resection/surgical resection followed by WBRT, 10.4 weeks (range, 7.5-13.3 weeks) for patients treated with GKS followed by WBRT/GKS/WBRT, and only 1 week (range, 0.0-3.3 weeks) for patients treated with only steroids. In multivariate analysis, surgical resection was significantly associated with longer survival compared to non-resection treatment (HR 0.23, 95% CI 0.08-0.66, p = 0.006). Interestingly, the patients with a shorter interval (≤12 months) from diagnosis of primary tumor to brain metastasis showed a longer survival time than the patients with a longer interval (>12 months) from diagnosis of primary tumor to brain metastasis, without statistical significance in multivariate analysis (15.6 weeks *vs* 8.7 weeks, p = 0.089). The younger age group (<60 years) survived longer than the older age group, but the difference was not statistically significant in multivariate analysis (14.8 weeks *vs* 8.7 weeks, p = 0.062).

**Figure 3:**
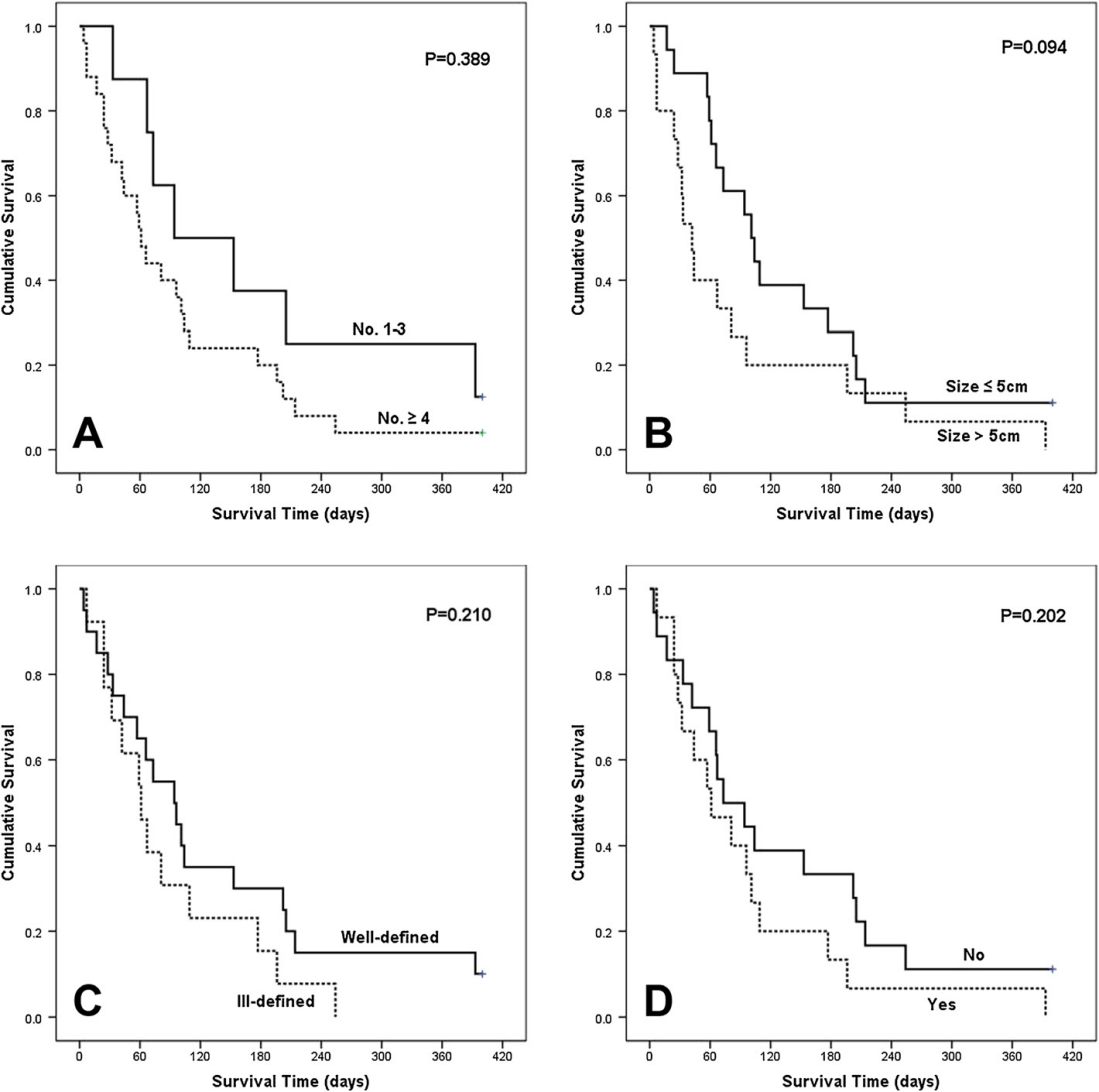
**Kaplan-Meier analyses of overall survival for 33 patients according to HCC characteristics.** (overall comparison was estimated using a log-rank test). **A**: Number of nodule, **B**: Size of the largest nodule, **C**: Type of HCC, **D**: Presence of portal vein thrombosis.

As shown in Figure 3, the univariate analysis demonstrated that HCC characteristics including the number of nodule, size of the largest nodule, type and presence of portal vein thrombosis were not correlated with the survival rate of the patients. In multivarate analysis, the patients without portal vein thrombosis survived longer than the patients with thrombosis did, with marginal statistical significance (10.4 weeks *vs* 8.7 weeks, p = 0.065). The group with well-defined HCC type or HCC nodule less than 4 showed a longer survival than the group with ill-defined type or nodule over than 4. However, these differences did not reach a statistical significance in multivariate analysis (HCC type [10.4 weeks *vs* 8.7 weeks, p = 0.072], number of nodule [9.6 weeks *vs* 8.7 weeks, p = 0.088]). Additionally, the usage of chemotherapeutic target agent (Sorafenib®) for HCC did not affect the patients’ survival (10.4 weeks *vs* 9.6 weeks, p = 0.583).

## Discussion

The incidence of brain metastasis in patients with HCC at our hospital was 0.65%, and this finding was in accordance with in previous reports demonstrating an incidence ranging from 0.26 to 2.2% [5,11]. However, these numerical values probably underestimate the true incidence. In our hospital, brain imaging study is not a routine evaluation for HCC patients; and it is performed mainly in cases with neurologic symptoms. Therefore, asymptomatic patients with brain metastasis may have been missed. In this study, only one patient (3%) was asymptomatic and found on a routine work-up for systemic progression.

The prognosis of brain metastasis from HCC is extremely poor. As summarized in Table 3, in the previously reported large studies, the median survival for patients with brain metastasis from HCC was from 4 to 12 weeks [5-7,12,13]. Similarly, the median survival was 10.4 weeks in our study. In a study by Han et al. [7], patients treated with WBRT and/or GKS had a longer median survival time than patients treated with surgical resection followed by WBRT (16 weeks *vs* 8 weeks). However, other studies have reported that surgical treatment may confer a survival benefit in patients with brain metastasis from HCC [5,6,13]. In our study, patients treated with surgical resection with/without postoperative WBRT had a longer median survival time than patients treated with GKS followed by WBRT/GKS/WBRT only or those treated with only steroids (25.3 weeks *vs* 10.4 weeks *vs* 1.0 week, p < 0.001). Furthermore, surgical resection was one of the most powerful prognostic factor for favorable survival in the multivariate analysis. (HR 0.23, 95% CI 0.08-0.66, p = 0.006).

**Table 3:** Previously published case-series in the literature of brain metastasis from HCC

**Author (year)**	**Patient number**	**Age (median)**	**Median interval between diagnosis of HCC and brain metastasis (months)**	**Median survival (weeks)**	**Treatment for brain metastasis**	**Survival time (weeks)**	**Positive prognostic factor**
Chang et al. (2004)	45	NA	10.5	4	Resection and/or radiotherapy	>16	Single lesion
					Supportive care	<4	
Choi et al. (2009)	62	54	18.2	6.8	Resection and WBRT	33.6	Single lesion, Child-Pugh’s classification A Any treatment modalities for brain metastasis
					Resection or WBRT or GKS	10	
					Steroids alone	2	
Hsieh et al. (2009)	42	55.8	15.4	4.8	NA	NA	ICH did not influence
Han et al. (2010)	20	55	18.5	8	Resection and WBRT	8	Younger age, Extracranial metastasis
					WBRT and/or GKS	16	
Jiang et al. (2012)	41	48.5	15	12	Resection or WBRT or GKS	18	No extracranial metastasis, Low RPA class, Any treatment modalities for brain metastasis
					Steroids alone	10.8	
Present Study (2013)	33	62	18.3	10.4	Resection/Resection+WBRT	25.3	No intratumoral bleeding Child-Pugh’s classification A, Resection for brain metastasis
					WBRT/GKS/WBRT+GKS	10.4	
					Steroids alone	1.0	

The treatment for intracranial metastasis from HCC may be similar to the general guidelines on metastatic brain tumors. In a single large lesion (>3 cm), especially with a significant mass effect (>1 cm midline shift), surgical resection should be considered with/without following whole brain radiotherapy (WBRT). Although the role of surgical resection for multiple brain metastases has not been established, surgery could be tried in cases with large lesions or significant mass effects and in cases where two or more lesions are accessible through a single craniotomy approach [14]. In the present study, it is difficult to conclude how the surgical resection could extend the patient’s survival. Surgical resection decreased the possibility of the death from neurologic origins (13% in surgical resection group *vs* 40% in radiation treatment group and 83% in palliative treatment group). This finding might be explained by the clinical course of hemorrhagic HCC metastasis. Han et al. [7] found that recurrent intracranial bleeding after treatment was frequently found in the patients who had presented with overt intratumoral hemorrhage. However, the patients who did better after surgical resection were likely the group of patients who had intracranial lesions in a less eloquent area or better overall prognostic factors or health status. This would confound the results in a view of treatment effect versus patient selection effect.

Child-Pugh classification was also an important prognostic factor in our analysis. Child-Pugh classification is the most commonly used criterion to evaluate the status of liver function. The rate of complications, hemorrhage and mortality increase with poor liver function, especially in patients with HCC or liver cirrhosis [15]. Choi et al. [6] reported that the Child-Pugh classification was shown to influence the median survival time in patients with brain metastasis from HCC. In our study, Child-Pugh classification A group had a longer median survival time than Child-Pugh classification B/C group (14.4 weeks *vs* 4.7 weeks, p = 0.038). On multivariate analysis, good liver function at the time of brain metastasis was one of the most powerful prognostic factor for favorable survival (HR 0.06, 95% CI 0.01-0.37, p = 0.002).

The median interval from initial HCC diagnosis to brain metastasis was 18.3 months in our study. It was similar to that in the previous studies, with the median interval ranging from 10.5 to 18.5 months. Some investigators, despite statistical insignificance, reported that the patients with a longer interval (≥12 months) from initial HCC diagnosis to brain metastasis had a relatively longer survival than patients with a shorter interval (<12 months) from initial HCC diagnosis to brain metastasis [6,13]. In our study, however, the patients with a shorter interval (<12 months) from diagnosis of primary tumor to brain metastasis showed longer survival than the patients with a longer interval (≥12 months) from diagnosis of primary tumor to brain metastasis without statistical significance in multivariate analysis (15.6 weeks *vs* 8.7 weeks, p = 0.089). Presumably, this result may be due to the declining general conditions of HCC patients. In other words, patients with a longer interval from diagnosis of primary tumor to brain metastasis might have worse general conditions following prolonged clinical progression than patients with a shorter interval from diagnosis of primary tumor to brain metastasis.

Previous studies have reported that brain metastasis from HCC is frequently associated with intratumoral hemorrhage [5-7,12,13]. This can be explained by the hypervascularity of HCC or a possible coagulopathy due to liver cirrhosis [6]. There is a controversy over the effect of intratumoral hemorrhage of brain metastasis on survival in HCC patients. Jiang et al. [13] reported that the patients without intratumoral hemorrhage had a longer survival time than patients with intratumoral hemorrhage, although statistical significance was not reached. Hsieh et al. [12], on the contrary, reported that the presence of intratumoral hemorrhage did not influence the overall survival in patients with brain metastasis from HCC. In our study, 51.5% of patients presented with intratumoral hemorrhage, and patients without intratumoral hemorrhage had a longer survival time than patients with intratumoral hemorrhage (13.7 weeks *vs* 8.1 weeks, p = 0.044 in univariate analysis). Additionally, the presence of intratumoral hemorrhage was the one of the prognostic factors for patient survival (HR 0.19, 95% CI 0.07-0.55, p = 0.002). As previously noted, hemorrhagic events frequently recurred in the cases with intratumoral hemorrhage, always drawn into the dismal prognosis. In addition, sudden neurological deterioration as in cerebrovascular accidents can lead to severe neurological deficits and poor functional status, which made the patients hesitating in selection for further adjuvant treatments, especially in the patients with underlying coagulopathy due to poor liver function state.

The RPA class has been suggested as an independent prognostic factor for the patients with brain metastasis from HCC. This result implies that patients with RPA class I and II may benefit from aggressive treatment [13]. Some studies revealed that the patient with low RPA class demonstrated longer survival than those with high RPA class [6,7]. Although patients with low RPA class (I and II) survived longer than patients with RPA class III in univariate analysis (13.4 weeks *vs* 2.4 weeks, p = 0.001) in our study, RPA class was not an independent prognostic factor in multivariate analysis (p = 0.575). The number of metastatic brain lesions can be an important prognostic factor. It was reported that the patients with single lesions had a longer survival time than the patients with multiple lesions [6]. Similarly, in our study, the median survival of patients with a single lesion was longer than that of patients with multiple lesions, although statistical significance was not reached (13.7 weeks *vs.* 8.1 weeks, p = 0.341). Han et al. [7] reported that younger patients less than 60 years of age can be considered as a positive prognostic factor. Our study also showed that younger patients survived longer than older patients with a marginal statistical significance (14.8 weeks vs 8.7 weeks, p = 0.096). However, younger age was not an independent favorable prognostic factor in the multivariate analysis (p = 0.062). Although good performance status (ECOG performance status grade 0–2) has been reported as a positive prognostic factor [6], it did not show statistical significance as a prognostic factor in our study. HCC characteristics including the number of nodule, size of the largest nodule, type and presence of portal vein thrombosis were not correlated with the survival time of the patients. Furthermore, these were not independent prognostic factors in multivariate analysis, despite marginal statistical significances in some factors (presence of portal vein thrombosis [p = 0.065], HCC type [p = 0.065], number of nodule [p = 0.088]).

## Conclusion

Although there have been some studies for identifying the prognostic factors for HCC patients with brain metastasis, it is challenging to specify the appropriate therapeutic strategy considering its rare incidence and poor prognosis. This study was basically a retrospective investigation of a relatively small number of patients. Therefore, there may be a possibility of selection bias and missed cases. And there was no information of the quality of life in the patients after various treatment modalities. To determine an appropriate therapeutic strategy for patients with brain metastasis from HCC, a further prospective randomized study based on the survival and quality of life is needed.

In addition to the treatment modality for brain metastasis and liver function at the diagnosis of brain metastasis, presence of intratumoral hemorrhage was an independent prognostic factors for survival. Although HCC patients with brain metastasis showed a very poor prognosis with a 1-year survival rate of 6%, surgical intervention was shown to lead to relative prolongation of the survival time, especially in those with preserved hepatic function.

## Abbreviations

CT: Computed tomography; GKS: Gamma knife radiosurgery; HCC: Hepatocellular carcinoma; MRI: Magnetic resonance imaging; WBRT: Whole brain radiotherapy.

## Competing interest

The authors declare that they have no competing interests.

## Authors’ contributions

MSH & KSM analyzed the data and drafted manuscript. KHL & SBC revised manuscript critically for important intellectually content. KHL & WYJ performed the statistical analysis. SHL & WYJ helped acquisition and interpretation of data. TYJ & IYK participated in reviewing literatures and helped in conception and design of the study. KSM & SJ conceived the study, participated in the design of it and coordination. All authors read and approved the final manuscript.

## Pre-publication history

The pre-publication history for this paper can be accessed here:

http://www.biomedcentral.com/1471-2407/13/567/prepub
